# Health-related quality of life of Southern Chinese with chronic hepatitis B infection

**DOI:** 10.1186/1477-7525-7-52

**Published:** 2009-06-05

**Authors:** Elegance TP Lam, Cindy LK Lam, CL Lai, MF Yuen, Daniel YT Fong, Thomas MK So

**Affiliations:** 1 Department of Medicine (Family Medicine Unit), The University of Hong Kong, 3/F, 161 Main Street, Ap Lei Chau Clinic, Ap Lei Chau, Hong Kong; 2 Department of Medicine, The University of Hong Kong, Queen Mary Hospital, Hong Kong; 3 Department of Nursing Studies, The University of Hong Kong, 4/F, William MW Mong Block, Faculty of Medicine Building, 21 Sassoon Road, Hong Kong; 4 Department of Medicine and Geriatrics, Princess Margaret Hospital, Hong Kong

## Abstract

**Background:**

Few studies have evaluated the health-related quality of life (HRQOL) of Southern Chinese with chronic hepatitis B (CHB) infection.

**Aim:**

To evaluate the HRQOL of Chinese patients at different stages of CHB infection and to find out factors associated with HRQOL.

**Methods:**

520 Chinese adult CHB patients of whom 156 were uncomplicated, 102 had impaired liver function, 139 had cirrhosis and 123 had hepatocellular carcinoma (HCC) were interviewed with a structured questionnaire, the SF-36 Health Survey version 2 (SF-36v2), and the Chronic Liver Disease Questionnaire (CLDQ). The differences in SF-6D health preference values and SF-36v2 scores between each CHB group and Hong Kong population norms were assessed by t-test. ANOVA was used to compare the mean SF-6D health preference, SF-36v2 scores, and CLDQ scores among CHB groups. Multiple linear regressions were performed to identify determinants of HRQOL.

**Results:**

CHB patients had significantly lower SF-36v2 scores than the population norm. The SF-6D values of CHB patients with uncomplicated disease, impaired liver function, HCC and cirrhosis were 0.755, 0.745, 0.720 and 0.701, respectively, all significantly lower than the population norm of 0.787. Advanced stage of CHB illness, anti-viral treatment, bilirubin level, psychological co-morbidity, younger age and female were associated with poorer HRQOL.

**Conclusion:**

CHB infection had a negative impact on HRQOL. There was a progressive decrease in health preference values with CHB disease progression. The results can be used for the estimation of quality adjusted life years (QALYs) for CHB patients in cost effectiveness or cost utility studies.

**Trial Registration:**

; HKCTR-151.

## Background

Hepatitis B virus (HBV) is the most common infection in the world. More than 2 billion people have been infected by HBV worldwide, of whom 350 million are chronically infected and more than one third (120 million) of them are in China [1]. An estimated 15–40% of chronic carriers may develop cirrhosis and hepatocellular carcinoma (HCC); resulting in over 1 billion people dying annually from hepatitis B related liver diseases [2]. The prevalence of chronic hepatitis B (CHB) is more than 10% in Southern Chinese including the population of Hong Kong [3]. Most chronic carriers in this region acquired the infections in the neonatal period or during early childhood [4], which means many people live with the threat of complications and the stigma of an infectious disease for many years.

Health-related quality of life (HRQOL) has become an important outcome indicator for chronic diseases in the last two decades. A number of studies have shown impaired HRQOL in patients with chronic liver diseases (CLD) including viral hepatitis, cirrhosis, cholestatic liver disease and HCC [5-13]. While there were several large studies on HRQOL of hepatitis C virus (HCV) patients [5,6,13], such data are scanty for CHB patients. Earlier studies suggested that patients with CHB infection had similar HRQOL as normal control [5,14,15], but the studies samples were small and selected, which limited the power and generalizability of the results [5,14]. Ong et al found that HRQOL measured by the SF-36 Health Survey and EQ-5D in Chinese asymptomatic CHB carriers was comparable to those of normal controls but cirrhotic and HCC patients had significant lower HRQOL scores [15]. Their study did not include a sufficient number of HCC patients for the differentiation between cirrhosis and HCC, and the effects of anti-viral treatment, duration of illness and clinical factors were not controlled for. Previous reports showed that disease severity [7,11,12,16], demographics [11,12], co-morbidity [6], and liver function biomarkers [10] could affect HRQOL in patients with CLD, but they have not been examined for Chinese CHB patients.

Apart from being an indicator of the health impact of CHB infection, a preference index converted from HRQOL can also be used for cost-effectiveness evaluation of interventions for CHB patients. A few studies have tried to rate the health preference of CHB infection by health care professionals or patients using disease-specific measures [17,18], but the results may not be valid because health preference should be measured by generic measures based on valuations by the general public, as recommended by the National Institute for Clinical Excellence (NICE), the United Kingdom [19]. Furthermore, HRQOL should be measured from the patient's perspective.

The aim of this study was to determine the HRQOL of patients at different stages of CHB infection and to find out factors associated with impairment of HRQOL, so that we can provide better health services to meet the needs of different CHB patient groups. We would like to establish the HRQOL preference values of different stages of CHB infection, which can be used for the calculation of quality adjusted life years (QALY) in cost-effectiveness and cost-utility analyses.

## Methods

### Subjects and data collection

The study was conducted from November 2006 to May 2008 in Hong Kong where 95% of the population is Southern Chinese. All subjects aged 18 years or above who were documented to be positive of hepatitis B surface antigen for more than six months were identified from the computerized registers of three public primary care clinics that had codings for CHB and recruited by clinicians from outpatient clinics of two regional hospitals that are the largest centers for CHB and HCC patients in Hong Kong. Written consents were obtained from all participants. We excluded patients who could not communicate in Cantonese; had severe cognitive impairment; co-infection with HIV, HCV, or hepatitis D virus, liver transplantation or end-stage non-hepatitis B related illnesses; were currently taking excessive alcohol (>30 units/week) or illegal drugs; or refused to give consent. Recruitment continued until there were at least one hundred patients in each CHB group.

All recruited CHB patients answered a structured questionnaire that comprised of the Chinese (HK [Hong Kong]) SF-36v2 Health Survey, the Chinese (HK) Chronic Liver Disease Questionnaire (CLDQ), and questions on socio-demographics and chronic co-morbidity administered by trained interviewers. Each patient was asked if he/she had ever been diagnosed by a registered medical practitioner for more than four weeks to have hypertension, diabetes mellitus, heart disease, stroke, chronic lung disease, arthritis, psychological illness (i.e. depression, anxiety, neurasthenia or psychosis) or any other chronic diseases. Chronic co-morbidity was measured by the total number of diseases (summation of positive responses to the questions) and the presence of a specific diagnosis. Clinical data related to the CHB infection including the use of anti-viral treatment, Child's staging for patients with cirrhosis, and the biomarkers of liver disease (Alanine Aminotransferase, Aspartate Aminotransferase, Alpha-fetoprotein and total bilirubin) of each patient were retrieved from the medical record. The duration of CHB illness was defined as the self-reported time from the first diagnosis by a registered medical practitioner to the day of the interview.

### Study instruments and outcome measures

#### The Chinese (HK) SF-36 Health Survey version 2 (SF-36v2)

The Chinese (HK) SF-36 Health Survey version 2 (SF-36v2) is a Chinese translation of the Medical Outcome Study (MOS) SF-36v2 Health Survey that has been validated and normed on the general Chinese population in Hong Kong [20,21]. The SF-36v2 Health Survey is a commonly used generic measure of HRQOL [22]. It measures eight scales including physical functioning (PF), role-physical (RP), bodily pain (BP), general health (GH), vitality (VT), social functioning (SF), role-motional (RE) and mental health (MH). Summations of item scores of the same scale give the scale scores, which are transformed into a range from 0 to 100, with higher scores indicating better quality of life [23]. The eight scale scores are aggregated into the norm-based physical and mental component summary (PCS and MCS) scores that have a population mean of 50 and standard deviation of 10.

#### The Chinese (HK) SF-6D

The SF-6D is a preference-based measure that can be mapped onto 11 items of the SF-36v2 Health Survey for the generation of a composite index value on a scale of 0 (death) to 1 (full health) [24]. It consists of six dimensions namely physical functioning (PF), role limitation (RL), social functioning (SF), bodily pain (PL), mental health (MH) and vitality (VT). The SF-6D scoring algorithm has been validated and established for the adult Chinese population in Hong Kong in previous studies [25,26]. The general population mean SF-6D preference value is 0.787, which was estimated from the SF-36v2 data of a general population survey of 2410 adult Chinese in Hong Kong in 1998 [20].

#### The Chinese (HK) Chronic Liver Disease Questionnaire (CLDQ)

The Chronic Liver Disease Questionnaire (CLDQ) developed by Younossi et al is a commonly used disease-specific HRQOL measure for liver diseases [27]. It consists of 29 items measuring six domains on abdominal symptoms (AS), fatigue (FA), systemic symptoms (SS), activity (AC), emotional function (EF) and worry (WO). Each item is rated on a 7-point Likert scale, with 1 indicating "all of the time" and 7 indicating "none of the time". Domain scores are calculated by the summated averages of endorsed item scores of the respective domains. An overall score is calculated by the mean of all domain scores. Each domain and the overall score range from 1 to 7, with a higher score indicating better HRQOL. The CLDQ has been translated into Chinese and shown to be a reliable and valid measure in Southern Chinese CHB patients in Hong Kong [28].

### Data analysis

Subjects were classified into four CHB groups: uncomplicated CHB, impaired liver function without cirrhosis or HCC, cirrhosis and HCC. Pearson's chi-square test was used to compare the distribution of socio-demographic variables between all CHB subjects and the general population [29,30]. One-way analysis of variance (ANOVA) or Pearson's chi-square tests were used to compare differences in socio-demographic and clinical characteristics among four CHB groups as appropriate. Continuous variables were tested by ANOVA and categorical variables were tested by Pearson's chi-square test. The SF-36v2 scores, SF-6D preference values and the CLDQ scores were calculated for overall and each of the four CHB groups. One-sample t-test was used to assess the difference in SF-36v2 scores and SF-6D health preference values between each CHB group and HK population norms. ANOVA was used to compare mean SF-6D, SF-36v2, and CLDQ scores among the four CHB groups. If significant differences were found by ANOVA, Dunnett's T3 tests were used to further examine the difference between individual CHB groups. Multiple linear regression analyses were performed to identify factors associated with lower HRQOL scores. Independent variables in the regression model were socio-demographics (age, sex, education attainment, marital status, occupation, monthly household income, smoking, drinking, and family history of CHB/CLD), chronic co-morbidities, and clinical factors (duration of illness, taking anti-viral treatment, stage of illness and liver function biomarkers).

All data analyses were carried out in SPSS for Windows 16.0. Statistical significant levels were set at p values less than 0.05.

## Results

A total of 879 CHB patients were invited for the study. 163 of them refused to participate (70 were busy; 40 did not give any reasons; 26 were not interested and 11 had health problems), and 86 patients (identified from the computerized registers) could not be contacted. Six hundred and thirty patients gave consent to the study, but 109 of them were excluded because they had one or more exclusion criteria. Five hundred and twenty patients completed the study (156 uncomplicated with normal liver function; 102 with impaired liver function; 139 with cirrhosis and 123 with HCC).

### Socio-demographic characteristics

Table 1 shows the socio-demographic characteristic of the study sample. The overall mean age of CHB patients was 50.4 ± 12.3 (SD) years old, and the majority were male (73.8%). CHB patients were more likely to be older, male, non-professionals and lower-income groups than the HK general population. There were no significant differences in socio-demographics among four CHB groups except that cirrhotic and HCC patients were older and more likely to be men than the other two groups since complications are more likely to develop in men and the median age for onset of complication is 57.2 years [31]. Drinking and smoking were more prevalent in CHB patients than the general population and in the three complicated CHB groups than the uncomplicated group.

**Table 1:** Socio-demographic characteristics of study subjects

	Uncomplicated CHB (n = 156)	Impaired LF (n = 102)	Cirrhosis (n = 139)	HCC (n = 123)	Overall (n = 520)	Population by-census and THS*
Age, yr (mean ± SD)‡	46.8 ± 12.6	44.6 ± 12.9	52.8 ± 9.3	57.0 ± 10.6	50.4 ± 12.3	NA
Sex (%)†‡						
Male	64.7	65.7	80.6	84.6	73.8	46.9
Female	35.3	34.3	19.4	15.4	26.2	53.1
Education attainment (%)‡						
Primary or below	24.4	16.7	26.6	37.4	26.5	25.4
Other education levels	75.6	83.3	73.4	62.6	73.5	74.6
Marital status (%)†						
Married	76.3	70.6	77.7	84.6	77.5	57.8
Other marital status	23.7	29.4	22.3	15.4	22.5	42.2
Occupation (%)†						
Administrative, managerial & professional	25.6	33.3	27.3	24.4	27.3	33.0
Other occupations	74.4	66.7	72.7	75.6	72.7	67.0
Household income (HK$, %)†‡						
<10000	28.8	26.5	47.5	46.3	37.5	27.9
10000–19999	21.8	20.6	19.4	17.9	20.0	27.8
20000–29999	25.6	14.7	10.1	11.4	16.0	17.4
>30000	16.0	27.5	13.7	14.6	17.3	26.9
Refused to answer	7.7	10.8	9.4	9.8	9.2	NA
Family history of HB or CLD (%)						
No	45.5	37.3	50.4	51.2	46.5	NA
Yes	54.5	62.7	49.6	48.8	53.5	NA
Smoking (%)†‡						
Never smoker	69.2	68.6	62.6	55.3	64.0	78.9
Former smoker	16.0	16.7	25.2	35.8	23.3	4.4
Current smoker	14.7	14.7	12.2	8.9	12.7	16.7
Drinking (%)†‡						
Never drinker	57.1	45.1	43.2	39.0	46.7	84.4
Former drinker	12.8	23.5	41.0	43.1	29.6	4.9
Current drinker	30.1	31.4	15.8	17.9	23.7	10.7

CHB = Chronic Hepatitis B; LF = Liver Function; HCC = Hepatocellular Carcinoma; HB = Hepatitis B; CLD = Chronic Liver Disease; NA; Not applicable.

*The 2006 Population by-census and Thematic Household Survey 2007 (Report No. 30) conducted by Census and Statistics Department, Hong Kong.

† Significant difference between CHB patients (overall) and general population (p < 0.05).

‡ Significant difference among the four CHB patients groups (p < 0.05).

### Clinical characteristics and co-morbid chronic illness

Table 2 describes the clinical characteristics and co-morbid chronic illnesses of CHB patients. The mean duration of CHB illness from diagnosis was 12.6 ± 9 (SD) years, with little difference between the groups. A higher proportion of patients in the cirrhosis group than others had ever taken anti-viral treatment. The prevalence of co-existing chronic diseases was significantly higher in patients with cirrhosis or HCC than other CHB groups, which may be an age effect.

**Table 2:** Clinical characteristics and co-morbid chronic illness of study sample

	Uncomplicated CHB (n = 156)	Impaired LF (n = 102)	Cirrhosis (n = 139)	HCC (n = 123)	Overall (n = 520)
Ever taken anti-viral treatment (%)†					
No	65.4	62.7	34.5	67.5	57.1
Yes	34.6	37.3	65.5	32.5	42.9
Liver function (%)†					
Normal LF	100.0	0.0	0.0	18.7	34.4
Impaired LF without cirrhosis	0.0	100.0	0.0	1.6	20.0
Cirrhosis					
Child-Pugh A	NA	NA	64.0	68.3	33.3
Child-Pugh B	NA	NA	17.3	8.1	6.5
Child-Pugh C	NA	NA	18.7	3.3	5.8
Duration of illness (mean, SD)	12.8 ± 8.1	12.3 ± 8.7	11.6 ± 8.5	13.6 ± 10.6	12.6 ± 9.0
Biomarkers					
ALT, U/L (mean, SD)†	27.4 ± 8.7	150.7 ± 306.5	48.2 ± 52.6	55.1 ± 47.3	67.8 ± 154.1
AST, U/L (mean, SD)†	24.1 ± 7.3	130.8 ± 272.0	48.2 ± 26.6	52.1 ± 38.8	57.4 ± 108.9
Abnormal AFP, ng/ml (%)*†	0.0	14.0	10.5	29.4	14.2
Bilirubin, umol/L (mean, SD)†	14.3 ± 7.0	20.1 ± 23.3	38.1 ± 56.5	18.4 ± 15.0	23.9 ± 35.3
Co-morbidity (%)					
Hypertension	23.7	17.6	19.4	26.0	21.9
Diabetes mellitus†	6.4	3.9	19.4	17.9	12.1
Heart disease	3.8	3.9	6.5	5.7	5.0
Stroke	0.0	1.0	1.4	0.8	0.8
Pulmonary disease	6.4	2.0	5.0	3.3	4.4
Joint disease	3.2	3.9	3.6	3.3	3.5
Psychological disease	4.5	4.9	3.6	8.9	5.4
Others	12.8	10.8	11.5	19.5	13.7
Any chronic illness†	38.5	31.4	51.8	53.7	44.2

LF = Liver Function; HCC = Hepatocellular Carcinoma; ALT, Alanine Aminotransferase; AST, Aspartate Aminotransferase; AFP, Alpha-fetoprotein.

Notes:

*Abnormal AFP refers to AFP value above 20 ng/ml.

† Significant difference among the four CHB groups (p < 0.05).

### Health-related quality of life (HRQOL) of CHB patients

Table 3 shows the mean SF-6D health preference values, SF-36v2, and CLDQ scores by CHB groups. The mean SF-6D and SF-36v2 scores of the normal general population are also shown for comparison. CHB patients, overall and by groups scored significantly lower than population norms in the SF-6D health preference values and nearly all SF-36v2 scores. The differences were the most substantial in the cirrhosis and HCC groups. It was surprising to find that the MCS scores of all CHB except cirrhosis groups were similar to that of the general population, and that the SF-36v2 GH and VT scores of the HCC group were a little higher than the population norm.

**Table 3:** Mean (SD) SF-6D, SF-36v2 and CLDQ scores by CHB groups

Scores (Norm)*	Uncomplicated CHB (n = 156)	Impaired LF (n = 102)	Cirrhosis (n = 139)	HCC (n = 123)	Overall (n = 520)	Significant difference
SF-6D						
Preference (0.787)	0.755† (0.14)	0.745† (0.15)	0.701† (0.15)	0.72† (0.16)	0.73† (0.15)	1>3‡
						
SF-36v2						
PF (90.6)	90.4 (13.3)	90.5 (13.0)	82.6† (16.0)	82.6† (16.6)	86.5† (15.3)	1>3, 1>4, 2>3, 2>4‡
RP (90.2)	85.2† (19.2)	79.7† (24.2)	68.8† (29.2)	70.7† (28.8)	76.3† (26.4)	1>3, 1>4, 2>3‡
BP (82.6)	72.9† (24.8)	70.2† (24.7)	70.3† (27.8)	71.1† (27.9)	71.2† (26.3)	NA
GH (53.2)	54.6 (20.5)	48.8† (20.7)	42.0† (22.5)	54.0 (22.1)	49.9† (22.0)	1>3, 3<4‡
VT (60.2)	65.1† (17.6)	62.4 (19.8)	55.4† (24.9)	61.6 (22.8)	61.2 (21.7)	1>3‡
SF (92.4)	86.3† (18.5)	82.0† (20.9)	73.7† (29.9)	74.4† (29.5)	79.3† (25.7)	1>3, 1>4‡
RE (88.5)	83.0† (18.6)	79.3† (21.9)	75.5† (26.6)	76.8† (26.4)	78.8† (23.6)	1>3‡
MH (72.0)	74.4 (16.1)	71.9 (20.3)	70.8 (19.3)	73.0 (20.3)	72.6 (18.8)	NA
PCS (48.8)	46.9† (9.2)	45.5† (9.6)	40.4† (11.2)	42.0† (11.5)	43.7† (10.7)	1>3, 1>4, 2>3‡
MCS (50.9)	50.7 (9.4)	48.6 (12.0)	47.3† (13.3)	48.9 (13.7)	49.0† (12.1)	NA
						
CLDQ						
AS	6.3 (0.9)	6.2 (1.0)	5.8 (1.4)	5.7 (1.3)	6.0 (1.2)	1>3, 1>4, 2>4‡
FA	5.3 (1.1)	5.0 (1.2)	4.7 (1.3)	4.9 (1.3)	5.0 (1.2)	1>3‡
SS	5.9 (0.9)	5.7 (0.9)	5.3 (1.1)	5.5 (1.1)	5.6 (1.0)	1>3, 1>4, 2>3‡
AC	6.3 (1.0)	6.0 (1.2)	5.6 (1.5)	5.7 (1.4)	5.9 (1.3)	1>3, 1>4‡
EF	5.6 (1.0)	5.3 (1.2)	5.1 (1.4)	5.2 (1.3)	5.3 (1.2)	1>3‡
WO	5.9 (1.2)	5.5 (1.3)	5.0 (1.7)	5.5 (1.4)	5.5 (1.5)	1>2, 1>3‡
Overall	5.9 (0.8)	5.6 (0.9)	5.3 (1.1)	5.4 (1.0)	5.6 (1.0)	1>3, 1>4, 2>3‡

CHB = Chronic Hepatitis B; LF = Liver Function; HCC = Hepatocellular Carcinoma; PF = Physical Functioning; RP = Role Physical; BP = Bodily Pain; GH = General Health; VT = Vitality; SF = Social Functioning; RE = Role Emotional; MH = Mental Health, PCS = Physical Component Summary Score; MCS = Mental Component Summary Score; AS = Abdominal Symptoms; FA = Fatigue; SS = Systemic Symptoms; AC = Activity; EF = Emotional Function; WO = Worry.

Notes:

*The mean scale and summary scores of adults >40 years old derived from the 1998 HK general population study.

† Significance difference between CHB groups (overall) and HK norm (p < 0.05).

‡ Significant difference between four CHB groups (p < 0.05). Group 1, uncomplicated CHB; group 2, Impaired LF; group 3, Cirrhosis; and group 4, HCC.

There was significant difference among the four CHB groups in the scale and summary scores of all three HRQOL measures (Table 3). Cirrhotic patients had the lowest scores, irrespective of the HRQOL measure used, among the four CHB groups. There was a progressive decrease in the mean SF-6D health preference values from 0.755 in the uncomplicated CHB group, to 0.745 in the impaired liver function group, 0.720 in HCC patients and 0.701 in the cirrhotics. The difference was statistically significant between the uncomplicated CHB or impaired liver function groups and the advanced complication (cirrhosis or HCC) groups. The difference between the uncomplicated CHB and the impaired liver function groups was not statistically significant. The only significant difference between these two groups was found in the CLDQ WO domain score. Although there was also no statistically significant difference in the SF-36v2 scores between the impaired liver function and uncomplicated CHB groups, the scores in several HRQOL domains (BP, GH, MH and MCS) of former group were close to those of patients with HCC.

### Determinants of HRQOL in CHB patients

Table 4 presents the results of multiple linear regression analyses of different HRQOL scores on the stage of CHB infection and other independent variables. The variables in the multiple regression models explained 23% to 29% the variances in HRQOL scores as indicated by the R-square.

**Table 4:** Multiple linear regression on HRQOL scores*

	SF-6D	SF36v2-PCS	SF36v2-MCS	CLDQ-Overall
	
	Coefficient (95% CI)	Coefficient (95% CI)	Coefficient (95% CI)	Coefficient (95% CI)
Stage of illness (vs. uncomplicated CHB)				
Impaired LF	-0.04† (-0.116, 0.034)	-3.80 (-9.083, 1.476)	-1.14 (-7.194, 4.914)	-0.44† (-0.94, 0.065)
Cirrhosis	-0.08†‡ (-0.143, 0.015)	-5.36‡ (-9.856, 0.869)	-2.59 (-7.742, 2.563)	-0.68†‡ (-1.106, 0.251)
HCC	-0.10†‡ (-0.16, -0.03)	-5.62‡ (-10.196, -1.045)	-3.70 (-8.944, 1.548)	-0.73†‡ (-1.161, -0.29)
Have taken treatment (vs. no treatment)	-0.04†‡ (-0.079, -0.003)	-1.84 (-4.536, 0.86)	-3.62†‡ (-6.71, -0.523)	-0.26†‡ (-0.516, -0.003)
				
**Clinical**				
Bilirubin (umol/L, 10^-2^)	-0.05 (-0.107, 0.008)	-7.38†‡ (-11.429, -3.337)	-4.33 (-8.968, 0.31)	-0.42†‡ (-0.807, -0.037)
				
**Co-morbidity**				
Psychological illness, present (vs. absent)	-0.10†‡ (-0.193, -0.015)	-4.36 (-10.643, 1.932)	-12.78†‡ (-19.993, -5.574)	-0.67†‡ (-1.272, -0.075)
				
**Socio-demographic**				
Smoking status (vs. never smoker)				
Former smoker	-0.06†‡ (-0.109, -0.017)	-3.24 (-6.491, 0.016)	-4.52†‡ (-8.248, -0.788)	-0.24 (-0.548, 0.071)
Current smoker	0.01† (-0.058, 0.074)	-3.21 (-7.854, 1.43)	4.46† (-0.862, 9.782)	0.23 (-0.212, 0.671)
Age (years, 10^-1^)	0.04†‡ (0.015, 0.056)	0.80 (-0.642, 2.239)	1.88†‡ (0.229, 3.532)	0.15†‡ (0.012, 0.286)
Sex, female (vs. male)	-0.07†‡ (-0.126, -0.015)	-5.73†‡ (-9.609, -1.859)	-2.08 (-6.528, 2.359)	-0.30 (-0.671, 0.066)
Occupation, others (vs. professional and administrative)	0.01 (-0.034, 0.057)	-3.45†‡ (-6.644, -0.259)	0.56 (-3.097, 4.225)	-0.14 (-0.443, 0.164)
Household income (vs. >30000)				
<10000	-0.07‡ (-0.127, -0.006)	-3.74 (-7.992, 0.503)	-3.90 (-8.766, 0.974)	-0.14 (-0.544, 0.264)
10000–19999	-0.03 (-0.095, 0.031)	0.40 (-4.038, 4.833)	-2.21 (-7.3, 2.872)	0.08 (-0.344, 0.5)
20000–29999	-0.01 (-0.076, 0.061)	-0.20 (-5.014, 4.621)	0.25 (-5.273, 5.774)	0.11 (-0.347, 0.569)
				
Constant	0.70 (0.582, 0.824)	52.63 (44.11, 61.146)	45.88 (36.118, 55.652)	5.63 (4.824, 6.444)
R-square	0.23	0.29	0.24	0.25

PCS = Physical Component Summary Score; MCS = Mental Component Summary Score; CHB = Chronic Hepatitis B; LF = Liver Function; HCC = Hepatocellular Carcinoma. Notes:

*Stage of illness, treatment, clinical, co-morbidities, and socio-demographic variables were entered as independent variables. Biomarkers (ALT, AST and bilirubin), duration of illness and age were treated as continuous variables; all other variables were entered as categorical variables. Sex, education attainment, marital status, occupation, taking anti-viral treatment, co-morbidity, and family history of HB/CLD and biomarker (AFP) were coded as dichotomous variables: female vs. male; primary or below vs. other educational levels; other marital status vs. married; other occupation vs. administrative, managerial and professionals, presence vs. absence of a diagnosis, abnormal vs. normal.

† Significant in overall model (p < 0.05).

‡ Significant difference compared with reference group which indicates in bracket, except for bilirubin and age (p < 0.05).

Stage of illness defined by the four CHB group classification was associated with SF-36v2 PCS, SF-6D and CLDQ overall scores. After controlling for liver function biomarkers and other confounders, compared with uncomplicated CHB, impaired liver function, cirrhosis or HCC were significantly associated with lower SF-6D and CLDQ overall scores. Stage of illness had no effect on the SF-36v2 MCS score, but taking antiviral treatment was negatively correlated with SF-36v2 MCS score. Higher bilirubin level was associated with lower scores in SF-36v2 PCS and CLDQ overall scores. Other liver function biomarkers such as ALT, AST or AFP did not have any significant effect on HRQOL.

Co-morbid psychological illness was negatively associated with all HRQOL scores, except the SF-36v2 PCS score. The effect of the total number of chronic disease or other specific chronic diseases did not reach statistical significance in the regression model.

A few socio-demographic factors had significant effects on HRQOL scores. Smoking was associated with lower SF-6D health preference values. Compared with never smoker, former smoking was also associated with worse SF-36v2 MCS score but paradoxically current smoking was associated with better SF-36v2 MCS. Increasing age was positively related to SF-6D health preference values, SF-36v2 MCS and CLDQ overall scores. Female was associated with lower SF-6D health preference values and SF-36v2 PCS scores. Compared with professional and administrative occupations, other occupations were associated with lower SF-36v2 PCS score. Lower income level was negatively related to SF-6D health preference values as compared with that high income level.

## Discussion

We used both generic and disease-specific measures to evaluate the HRQOL of CHB patients in this study. The generic measures allowed the comparison with the normal population. The disease-specific CLDQ was more sensitive and addressed some important HRQOL domains (e.g. worry) specifically associated with this disease. A significant difference between patients with uncomplicated CHB and impaired liver function was found only in the CLDQ WO domain, but in none of the SF-36v2 scale or SF-6D scores, indicating higher sensitivity of the former measure. The disease-specific and generic measures are recommended for the evaluation of the effect of CHB infection and its treatment on HRQOL. We also used the SF-6D to convert HRQOL to a composite preference index that could quantify and rank the quality of life impact of each stage of CHB infection.

### Comparison with HK general population norm

We found all CHB patients including those without any complications had significantly lower SF-36v2 and SF-6D scores than the population norms. Overall the effect size differences were around 0.4, which corresponded to the minimal important differences of 0.3 to 0.5 commonly found with HRQOL measures [32,33]. The study by Ong et al found CHB patients with abnormal liver function had only modest impairment of HRQOL [15] but we found that patients with impaired liver function had significant impairment in several HRQOL domains (SF-36v2 BP, VT, MH and MCS) approaching the level of HCC patients. Previous studies reported that uncomplicated CHB without impair HRQOL might have underestimated the HRQOL effect by comparing to controls recruited from tertiary health care centers who might have other illness that impaired HRQOL [5,14,15]. The choice of HRQOL measures could also affect the sensitivity in detecting any difference.

The findings did not support our hypothesis that uncomplicated CHB caused significant mental stress because the SF-36v2 MCS score was near normal in all except the cirrhotic patients. It was unexpected that SF-36v2 GH and VT scores in patients with HCC were higher than the population norms. There are a few possible explanations. Firstly, many HCC patients in this study were in remission after surgical resections. Some studies have shown significant improvement of HRQOL in patients with HCC after hepatic resection at three months [34,35]. Secondly, adaptation and positive coping behaviours might have led to a response shift in HCC patients' HRQOL perception. They may become more optimistic towards their illness especially after successful treatment, and they often adopt healthier lifestyles such as doing more exercises to improve their quality of life. Thirdly, family and social support given to cancer patients may also improve HRQOL [36].

### Comparison among CHB groups

We observed a decrease in the mean SF-6D health preference values along the progressive stages of CHB infection from 0.755 in uncomplicated patients to 0.745 in those with impaired liver function to 0.720 in HCC patients and 0.701 in cirrhotics. Previous studies have shown the minimal important difference of the SF-6D preferences value ranged from 0.01 to 0.048, with a weighted mean estimate of 0.03 [37]. Therefore the differences between the cirrhotic (0.054) and HCC (0.035) groups and the uncomplicated group were probably important. Levy et al also reported a significant drop in the health preference values measured by a disease-specific measure from 0.68 in uncomplicated CHB infection to 0.38 in HCC patients and 0.35 in decompensated cirrhosis [38]. The health preference values reported by Levy et al were much lower than those measured by the SF-6D in our study. One possible explanation was that disease-specific measures as that used in Levy's study might over-estimate the negative impact of disease by focusing on impairments and symptoms related to the disease. Differences in the subjects and methods of preference measurement can also lead to a discrepancy in results. Levy's study sampled both CHB patients and uninfected persons to generate the preference values using hypothetical health states, while our study measured the SF-6D preference values of the actual HRQOL states of the different CHB patient groups.

Cirrhotic patients had the worst HRQOL scores measured by both generic and disease-specific measures. Cirrhotic patients may suffer from complications such as ascites, variceal bleeding and hepatic encephalopathy that can severely impair HRQOL [8]. They also often have symptoms such as fatigue, anorexia and weight loss that lead to limitation in daily functioning, loss to work and financial difficulty. The difference between cirrhotic and HCC patients did not reach statistical significance in all except the SF-36v2 GH scores, which were consistent with the findings from Ong et al study [15].

Patients with impaired liver function had lower SF-6D health preference values and SF-36v2 scores in all scales than the uncomplicated group although the differences did not reach statistical significance. They had significantly lower CLDQ WO score than the uncomplicated CHB group, probably because they were more worried about cirrhosis or HCC.

### Determinants of poor HRQOL

The association between more advanced stages of CHB illness with worse HRQOL persisted after controlling for confounding variables, which was consistent with the findings from other studies [15,16]. Biomarkers such as ALT and AST had no effect on HRQOL, although they are often used as a guide to anti-viral treatment. It was interesting to find that taking of anti-viral treatment had negative effect on the SF-36v2 MCS score. This could be due to side effects of treatment or the selection of the patients who were more ill or anxious for treatment. Previous studies on HCV patients also found that anti-viral drug treatment reduced HRQOL initially [39] but an improvement was observed after successful eradication of the virus [40,41]. Further longitudinal studies are needed to determine the causal relationship between anti-viral treatment and HRQOL.

Previous studies found older age was associated with poorer HRQOL in CLD patients [12,16], but our study showed that age actually had a positive effect, after controlling for the liver disease status and co-morbidities. This shows the importance of controlling for confounding variables in HRQOL data analysis. Females have lower HRQOL scores than males, as shown in other studies [12,16]. Females tend to be more likely to worry about their illness, and they have lower HRQOL scores in general [42].

## Limitations

It was a cross-sectional study and we could not confirm the causal relationship between anti-viral treatment and HRQOL. Subjects in this study were a convenient sample that might not be fully representative of all Southern Chinese but we could not identify any systematic bias in our results. Patients were all Southern Chinese, so the results might not be generalizable to other ethnic groups.

## Conclusion

The health-related quality of life (HRQOL) of Southern Chinese adult patients with chronic hepatitis B (CHB) infection were significantly lower than that of the general population even among those without any biochemical or clinical complications. CHB infection was associated with an overall reduction of 0.057 in SF-6D preference value from the population norm. The presence of advanced complications such as cirrhosis or HCC was the most significant negative determinant of HRQOL in patients with CHB infection. Anti-viral treatment, bilirubin level, co-morbid psychological illness, younger age and female were also associated with poorer HRQOL. The SF-6D preference values dropped from 0.755 in uncomplicated CHB patients to 0.701 and 0.720 in cirrhotic and HCC patients, respectively. The preference values of the different stages of CHB infection can be used for the estimation of quality adjusted life years for the respective patients in cost effectiveness and cost utility studies.

## Competing interests

The authors declare that they have no competing interests.

## Authors' contributions

All authors participated in the design of the study; ETPL, CLL, MFY and TMKS collected the data; ETPL, CLKL and DYTF were involved in data analysis and interpretation; and ETPL and CLKL drafted the manuscript. All authors read and approved the final manuscript.
